# Phenotyping Alfalfa (*Medicago sativa* L.) Root Structure Architecture via Integrating Confident Machine Learning with ResNet-18

**DOI:** 10.34133/plantphenomics.0251

**Published:** 2024-09-11

**Authors:** Brandon J. Weihs, Zhou Tang, Zezhong Tian, Deborah Jo Heuschele, Aftab Siddique, Thomas H. Terrill, Zhou Zhang, Larry M. York, Zhiwu Zhang, Zhanyou Xu

**Affiliations:** ^1^ United States Department of Agriculture—Agricultural Research Service—Plant Science Research, St. Paul, MN 55108, USA.; ^2^ Department of Agronomy and Plant Genetics, University of Minnesota, St. Paul, MN 55108, USA.; ^3^ Department of Geography and Geology, University of Nebraska at Omaha, Omaha, NE 68182, USA.; ^4^ Department of Crop and Soil Sciences, Washington State University, Pullman, WA 99164, USA.; ^5^ Department of Biological Systems Engineering, University of Wisconsin, Madison, WI 53706, USA.; ^6^ Department of Agricultural Sciences, Fort Valley State University, Fort Valley, GA 31030, USA.; ^7^ Biosciences Division and Center for Bioenergy Innovation, Oak Ridge National Laboratory, Oak Ridge, TN 37830, USA.

## Abstract

**Background:** Root system architecture (RSA) is of growing interest in implementing plant improvements with belowground root traits. Modern computing technology applied to images offers new pathways forward to plant trait improvements and selection through RSA analysis (using images to discern/classify root types and traits). However, a major stumbling block to image-based RSA phenotyping is image label noise, which reduces the accuracies of models that take images as direct inputs. To address the label noise problem, this study utilized an artificial intelligence model capable of classifying the RSA of alfalfa (*Medicago sativa* L.) directly from images and coupled it with downstream label improvement methods. Images were compared with different model outputs with manual root classifications, and confident machine learning (CL) and reactive machine learning (RL) methods were tested to minimize the effects of subjective labeling to improve labeling and prediction accuracies. **Results:** The CL algorithm modestly improved the Random Forest model’s overall prediction accuracy of the Minnesota dataset (1%) while larger gains in accuracy were observed with the ResNet-18 model results. The ResNet-18 cross-population prediction accuracy was improved (~8% to 13%) with CL compared to the original/preprocessed datasets. Training and testing data combinations with the highest accuracies (86%) resulted from the CL- and/or RL-corrected datasets for predicting taproot RSAs. Similarly, the highest accuracies achieved for the intermediate RSA class resulted from corrected data combinations. The highest overall accuracy (~75%) using the ResNet-18 model involved CL on a pooled dataset containing images from both sample locations. **Conclusions:** ResNet-18 DNN prediction accuracies of alfalfa RSA image labels are increased when CL and RL are employed. By increasing the dataset to reduce overfitting while concurrently finding and correcting image label errors, it is demonstrated here that accuracy increases by as much as ~11% to 13% can be achieved with semi-automated, computer-assisted preprocessing and data cleaning (CL/RL).

## Introduction

### Description of alfalfa and its importance

Alfalfa, also known as lucerne (*Medicago sativa* L.), is the third most widely produced crop in the United States [1] and yielded over 49.2 million tons of harvested material from over 6.1 million hectares in 2021 [1]. The global dairy and beef industries rely heavily on alfalfa to sustain them. Subsequently, genetic improvements in alfalfa have a direct and marked effect on beef and dairy industry outputs. Additionally, alfalfa benefits the global food production ecosystem and accounts for one-third of U.S. honey production [2]. Alfalfa root systems provide several ecosystem services such as atmospheric carbon (CO_2_) and nitrogen (N) fixation. N is fixed via a symbiotic relationship between roots and nitrogen-fixing soil bacteria [3], therefore amending and replenishing depleted soils (especially from monoculture). Additionally, the especially deep growth habit of alfalfa roots (6 to 15 m) [4,5] functions differently compared to other shallow-rooted plants (such as maize [*Zea mays* L.] or soybean [*Glycine max* L.]) by sequestering atmospheric C deep in soils via a natural and low-cost process. Despite being the oldest crop grown solely for forage [6] and the major role of alfalfa in various steps in food production, it has received less-than-adequate attention in investigating the root structure and genetics research to improve crop quality and increases in crop yields, compared with other crops such as maize, soybean, or wheat (*Triticum aestivum* L.). Alfalfa root system architecture (RSA) breeding has shown that branch and taproot RSAs can be maintained across different environments and nutrient supplies [7], that increased herbage yield in alfalfa can be associated with selection for fibrous and lateral roots [8], and that deep taproots are associated with drought tolerance and high winter survival rates. Divergent selection and breeding for branch and taproot RSAs have proven successful [9] for alfalfa RSA improvement, and this research continues the use of the resultant classification and class labels with the terms “Branched”, “Taproot”, and “Intermediate”.

### RSA research and image analyses using AI techniques

The foundation of imagery-based RSA research is rooted in applying non-artificial intelligence (AI) computer vision (CV) methods to investigate images derived from RGB cameras and scanners; however, there is a growing trend in RSA research that employs AI-driven image analysis techniques to detect, classify, and quantify plant traits important to breeders and producers. This trend is spurred by the confluence of several technologies, including but not limited to in silico AI methods that mimic human intelligence, machine learning (ML), deep learning (DL), high-resolution digital imaging sensors (hyperspectral cameras and tomographic sensors), robotic automated processes, and supercomputers capable of efficiently processing large amounts of data. The coupling of these technologies and methods provides partial solutions to the challenges and limitations of manual data collection and plant trait sampling (selections/phenotyping) in that in silico trait analysis is quicker, more accurate, and more capable of handling large amounts of data.

In general, there are 2 approaches to detecting and classifying RSA from images while also employing AI methods. The first approach involves capturing images and then extracting root features from those images. For example, an alfalfa root crown image is used as input into feature extraction software such as RhizoVision Explorer [10]. Outputs of the feature extraction process can include trait measurements regarding topology, morphology, and distribution. These root traits/features are then used as inputs into AI-based models such as decision trees (i.e., Random Forest [RF]) that classify/label those images based on the input features. The second approach does not utilize feature extraction to classify the RSA. Instead, this method uses the image (pixels) as direct input into the AI model and, through training of the model with labeled images, tunes the model to “learn” how to correctly label new images based on its training.

Despite the fledgling nature of RSA research that couples AI and digital image processing, there has been some progress achieved in the last decade focusing on alfalfa RSA. Some authors investigated trait heritability of alfalfa seedlings with images using RF and Gradient Boosting Machine ML algorithms and found that plants as young as 2 weeks in age can be effectively used for phenotypic root trait selections (a rapid selection process) [9]. Other authors studying alfalfa RSA found that the RF model had the highest balanced accuracies compared to several other tested models and that image augmentation can have marked positive effects on the prediction power of models with dissimilar architectures/methods [11]. Utilizing an alfalfa root imagery dataset from another group [12] to classify based on the WCSMO (Water Cycle Spider Monkey Optimization)-based deep convolutional neural network (CNN), their optimized model achieved higher sensitivity, specificity, and accuracy scores than the other models they compared with the same dataset [13].

#### Image labeling

Regardless of the model(s) employed for RSA analysis, the quality of the image data labels is a limiting factor for any research in that the performance of the model is inhibited by poor data. Classification or prediction accuracy is partially dictated by the accuracy of the image labels because image data labels are the first major stumbling block to model performance. Many ML/AI models require both image data (pixels) and image metadata (labels) to perform classification and prediction tasks. Therefore, efforts to create clean data and/or efforts to cleanse inaccurate data labels (noisy labels) are often performed prior to executing the subsequent image analysis models. One method to reconcile image data label issues is to manually relabel problematic images; however, it usually increases labor as some relabeling requires human interventions in an otherwise in silico process. For images with multiple regions of interest (ROIs) or objects, objects can be identified with bounding boxes where each gets its own specific label based on the content identified within each bounding box. For large image datasets or images with multiple ROIs and objects, manual labeling is time-consuming, error prone, and costly [14–16].

Semi-automatic image labeling systems offer partial solutions to the manual labeling problems such as low-speed label generation, human labeling errors, and the high cost of labeling by partially automating image annotation. These labeling systems include an unsupervised feature-learning procedure (feature extraction), semi-supervised label estimation (propagate labels with high confidence), and a visual analysis step that interactively propagates labels from less-confident samples [14,17,18]. Each type of semi-automatic system addresses the label estimation step a little differently about querying human operators about unlabeled data. However, the systems still query regarding some unlabeled images.

Automatic image labeling is the fastest annotation method and involves using software to add labels to images in silico without human input or intervention. The simplest method is batch labeling, where simple programming assigns names to imagery files in a directory. Batch labeling requires labels that are generic and/or non-specific to each image’s content [19]. However, this method does not extract features. A computational method, reactive machine learning (RL), was developed to address the problem of determining which data should be actively labeled or relabeled to optimize model classifications [20]. RL is defined as a generalization of active learning that explores the trade-off between decreasing the noise (incorrect labels) of a training dataset by relabeling and increasing the size of the noisier dataset by labeling new examples. The algorithms contain 2 distinct methods: (a) impact sampling that identifies the labeled images (also called examples) that have the highest potential to change the classifier, and (b) an extension of uncertainty sampling that aggregates the uncertainties of both the classifier and the dataset labels (a weighted average of the 2 uncertainties) [20]. The question of whether it is possible to create an intelligent active learning approach capable of automatically querying “appropriate” examples (annotated images in a dataset) for new labels (relabeling) to achieve the highest accuracy classifiers possible for a given labeling budget was posed [21]. A partial answer was the creation of a method for estimating uncertainty in dataset labels, called confident machine learning (CL) [22]. The approach uses image label data to characterize and identify label errors in datasets by pruning, counting, and training with confidence [22].

### Challenges of RSA research

While progress has been made regarding the image-based and model-driven characterizing and classification of alfalfa RSA, there are several image analysis challenges after acquiring the root images. The first challenge is that some models need predefined RSA traits. Bucciarelli et al. [9] and Xu et al. [11] extracted 38 variables (including branch number, length, area, volume, etc.) using the commercial tool WinRHIZO to analyze the roots and used the extracted variables as predictors to develop ML and DL models. Similarly, Mattupalli et al. [12] extracted 38 variables by scanning the images with the platform RhizoVision Crown and studied the relationship between various root features extracted using RhizoVision Analyzer. Predefined features from the root images have been proven as an option to study root traits; however, the required human inputs to predefine a limited number of known traits can incorporate bias and errors into the data depending on the methods and/or tools used for feature extraction (such as root tracing or node selection that relies on human inputs, or segmentation models that require human annotations). In contrast, using all the pixels from root images without human inputs allows the CV and DL models to find the individual and combinations of the most informative pixels. This image-direct method has the potential to improve the prediction accuracy, increase throughput, and identify patterns and rules for root breeding thus far undetectable by humans. The second challenge for the RSA research is acquiring the ground truth of the root traits, especially from destructive sampling and collecting images. All supervised ML requires ground truth to train the model, but RSA traits are affected from growing environments (E), have large gene by E interactions, and different RSA researchers may rate the roots differently. RL and CL have proven effective to improve labeling accuracy as evidenced in Northcutt et al. [23] and Lin et al. [20], thus helping to obtain the ground truth and subsequently improving model performance via increased prediction accuracies.

The aim of this study was to test the ability of combining the RL and CL methods with the ResNet-18 deep convolutional neural network (DNN) model to circumvent the ground-truth challenge of accurately detecting root traits within the model and thereby increase the prediction accuracies of classifying 3 different RSAs of alfalfa root images from 6 different populations by using images as direct model inputs that help to remove human bias/errors caused by subjective feature extraction processes. Additionally, this study highlights the moderate abilities of 2 separate label correction strategies to improve model outputs (increasing the percentage of correctly predicting RSA for unlabeled root images), as well as the ability of the ResNet-18 model to reduce human errors or bias by reducing subjectivity in the modeling process via image-direct inputs to the model.

## Materials and Methods

### Experimental design and plant materials

**Fig. 1.**
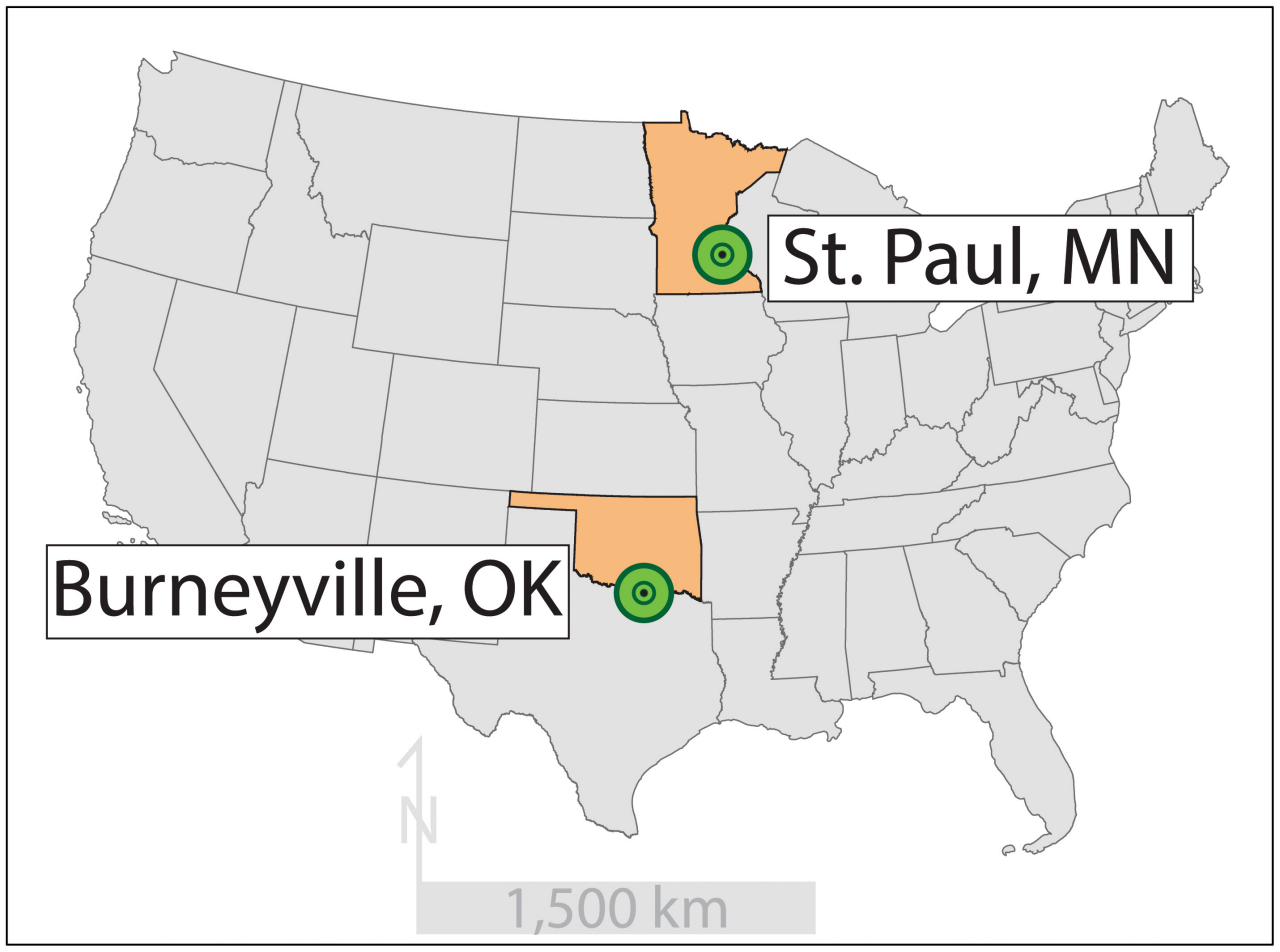
Sample locations.

Two sets of alfalfa root images from separate experiments were used as input data for this research. The first dataset with 617 images was acquired (with permission) from plants grown in St. Paul, MN [11] and the second dataset with 264 images was acquired (with permission) from plants grown in Burneyville, OK [12] (Fig. 1). The Minnesota dataset primarily focuses on phenotypic variations within specifically selected populations, whereas the Oklahoma dataset highlights the environmental and pathological factors influencing root morphology.

**Fig. 2.**
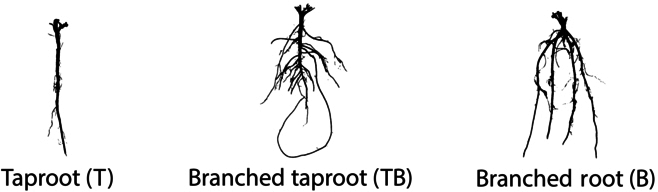
Root phenotypes from UMN4561 and UMN4563 fourth cycle progenies.

The St. Paul, MN dataset was produced from 5 alfalfa populations that were created from phenotypic selections for RSA types starting from a parental unselected population UMN2892 [11]. Two populations were the result of 3 cycles of phenotypic selection for branch type (UMN3233) and taproot type (UMN3234) (Fig. 2). Plants selected from these 2 populations were then randomly intermated to produce progenies (fourth cycle) that were evaluated for the desired root phenotypes (UMN4561 from UMN3233 for branch type; UMN4563 from UMN3234 for taproot type). The resultant 5 populations were hand seeded in plots (1.4 m × 0.9 m) with 28 equally spaced (13 cm × 13 cm) plants per plot. Six replicate plots per population were randomly spaced throughout the field and were surrounded by a border row of the alfalfa cultivar “Agate”. Seeding was performed at the University of Minnesota St. Paul Experiment Station (Fig. 1) on 2016 June 1 into Waukegan fine-silty loam: a sandy-skeletal, mixed, superactive, mesic Typic Hapludoll. Plants were controlled for pests and weeds and were irrigated as needed. Plants were clipped to 3 inches in the fall (September 2016). The plant root crowns were harvested at a depth of 30 cm using the shovelomics method [24] at 22 weeks after seeding on 2016 October 12. The roots were washed to remove soil, the foliage was removed 4 cm above the root crown, and then roots were stored at 4 °C prior to image acquisition.

The Burneyville, OK image dataset was created from a commercial hay production field containing the alfalfa cultivar America’s Alfalfa Alfagraze 600 RR [12]. The sample location (Noble Research Institute’s Red River Farm—Burneyville, OK) (Fig. 1) soils are categorized as Gaddy loam to Yahola fine sandy loam [12]. Plants (264 total) were dug in October 2017 at the onset of dormancy and were sampled from 3 populations of varying levels of Phymatotrichopsis root rot (PRR) disease caused by *Phymatotrichopsis omnivora*: (a) dead plants at the disease front (24 plants), (b) a zone of mostly dead but containing some survivor plants (120 plants), and (c) an asymptomatic zone with no visible signs of disease (120 plants). Root crowns were extracted from soil using the shovelomics method at a depth of 20 to 25 cm. Soil residue was removed from the roots via brushing.

### Image acquisition, preprocessing, and labeling

**Fig. 3.**
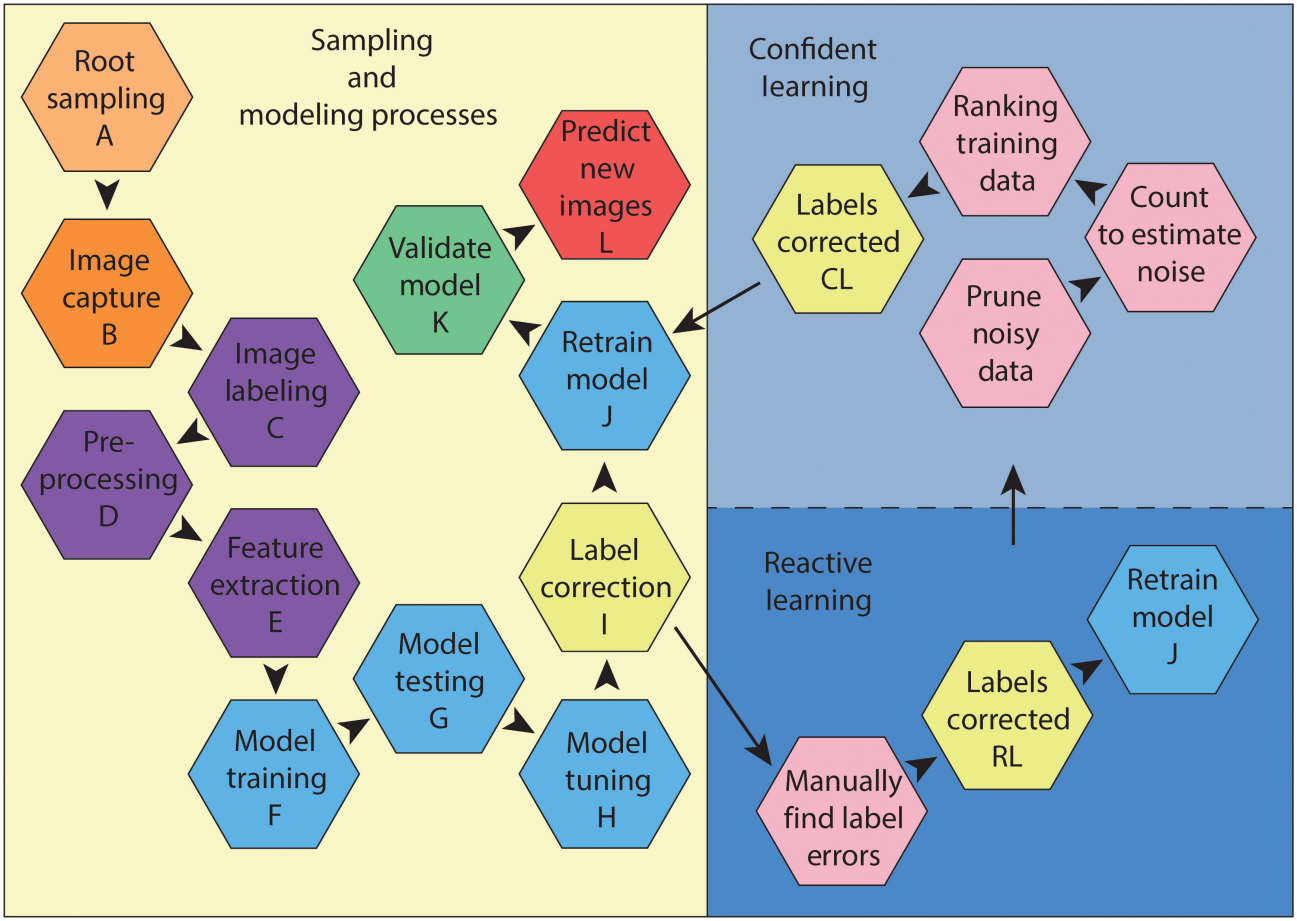
Workflow for sampling and imaging RSA of alfalfa root crowns, image label creation and correction, and model implementation. Orange represents field or lab sampling; purple represents image preparation methods; blue represents modeling methods; yellow represents image label correction process; pink represents image label correction steps; green represents model validation on new datasets and predictions for unlabeled data; red represents model predictions on new dataset images.

There are numerous procedures and methods involved in the process of RSA image analysis. Figure 3 diagrams the main steps of this workflow.

#### Image acquisition

**Fig. 4.**
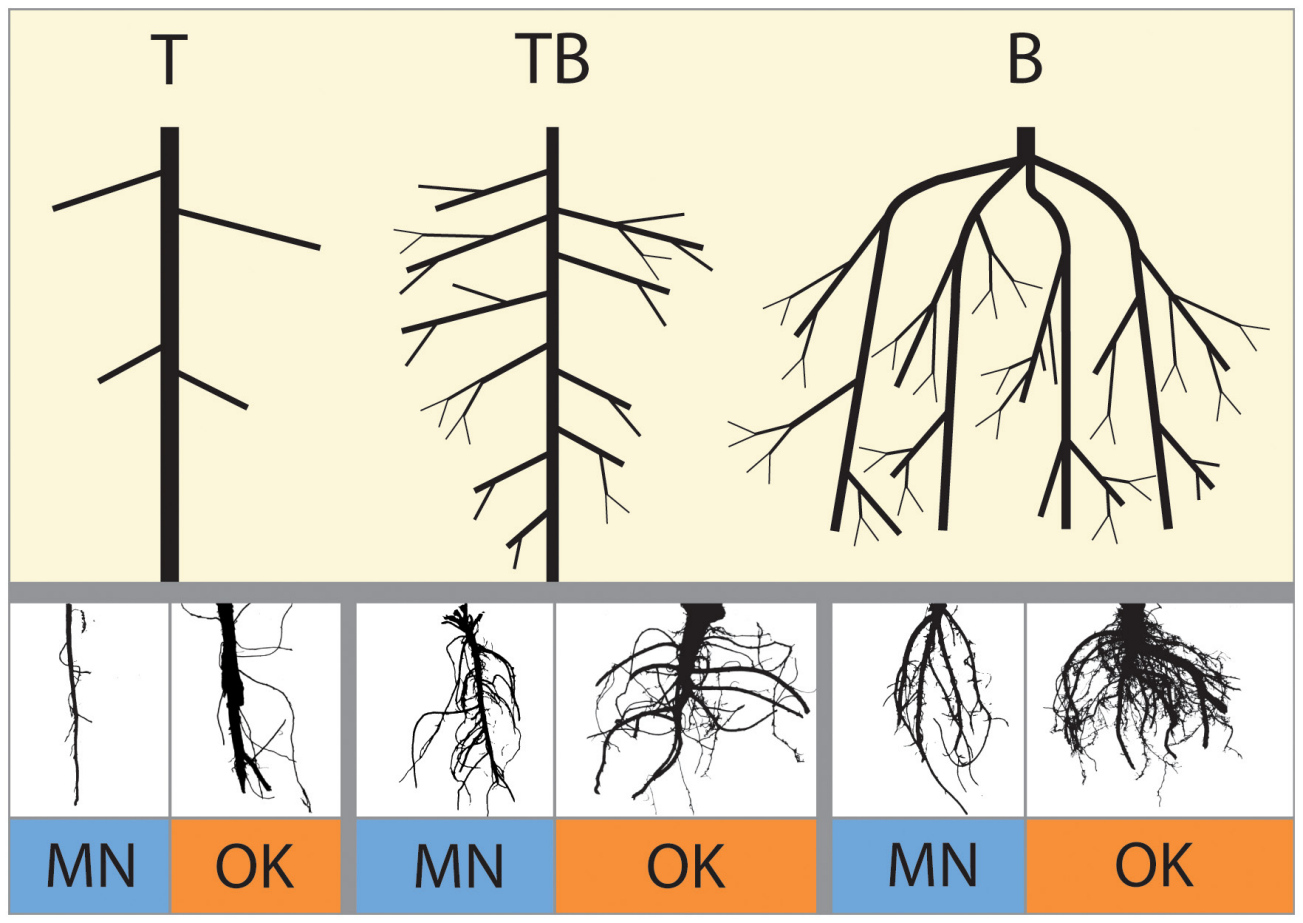
Segmented sample root images and synthetic idealized root system architectures. Top row shows simulated roots. Bottom row shows sampled roots from both study locations. “T” represents taproot RSA. “TB” represents intermediate RSA. “B” represents branched RSA. Scale is not consistent between sample root images.

Image data generated from Minnesota [11] (617 images) was created in the laboratory using an RGB DSLR camera (Panasonic DMC-FZ30, Newark, NJ). These images were subsequently post-processed to remove scale bars and ID tags with ImageJ [25], then segmented into fore- and background pixels resulting in binary images using RootPainter software [26] (Figs. 2 and 4). The segmented images were then labeled by 3 of the authors using a common protocol [9] to differentiate the 3 root types (taproot-dominant T, branching-dominant B, intermediate-branching taproot TB) (see Fig. 4) in each dataset, with the final image label for each root decided using the majority rule [21].

Root images from the Oklahoma [12] plants were obtained using the RhizoVision Crown platform that employed a monochrome machine vision camera (Basler acA3800-um, Graftek Imaging, Inc., Austin, TX) [27]. The images were then segmented (into binary PNG images) using RhizoVision Analyzer [28] and subsequently labeled by the current authors into 3 different classes with the same method used for the Minnesota [11] images (same 3 classes T, B, and TB as the Minnesota dataset).

#### Feature extraction and topology data

Feature and topology data of both the Minnesota and Oklahoma images were obtained by the free and open-source software RhizoVision Analyzer (Oklahoma data) and its modern replacement, RhizoVision Explorer v2.0.2 [10,29] (Minnesota data). A total of 38 (Minnesota) and 27 (Oklahoma) predefined topological/feature traits were extracted from the original (non-augmented) sample images by scanning the root images. For the Minnesota [11] dataset, these traits (such as lengths, areas, branching number, etc.) were used as input to construct the multiple RF decision trees. The methods (and RhizoVision Explorer/Analyzer settings) to extract these topological traits and trait names can be found in Xu et al. [11] and Mattupalli et al. [12].

#### Root labeling protocol

Root labels were created based in part on the RSA labeling protocol of Bucciarelli et al. [9]. In brief, taproots (T) were defined as having less than 4 lateral roots emerging from the taproot (primary growth) that were spaced 3 to 4 cm apart, branched (B) roots were defined as producing 4 to 6 thick lateral roots along the taproot at 1- to 2-cm intervals, and intermediate roots (TB) were defined as having 4 or more lateral roots spaced more than 2 cm apart and are neither T nor B types.

#### Image segmentation

For both datasets, the binary images’ black root pixels and white background pixels were inverted to obtain white root tissue pixels on a black background. Subsequently, the root tissue pixels were extracted using a maximum bounding box that covered all roots and were then padded to create a square with black pixels, ensuring that root tissue dominated the binary image.

#### Image augmentation

To increase image sample size and reduce the risk of overfitting due to overparameterization [30,31], the dataset images were augmented using a variety of common image augmentation techniques. Augmentation included vertical and horizontal flipping, image rotating (between −180° and 180°), and random image scaling/resizing (from 0.5 to 1.5 times the original size). All augmentation python scripts were implemented using the “transforms” function from the *torchvision* package (https://github.com/pytorch/vision). The process generated 10 times the original amount of initial data (881*10 images).

### Model selection, parameterization, and label corrections

**Fig. 5.**
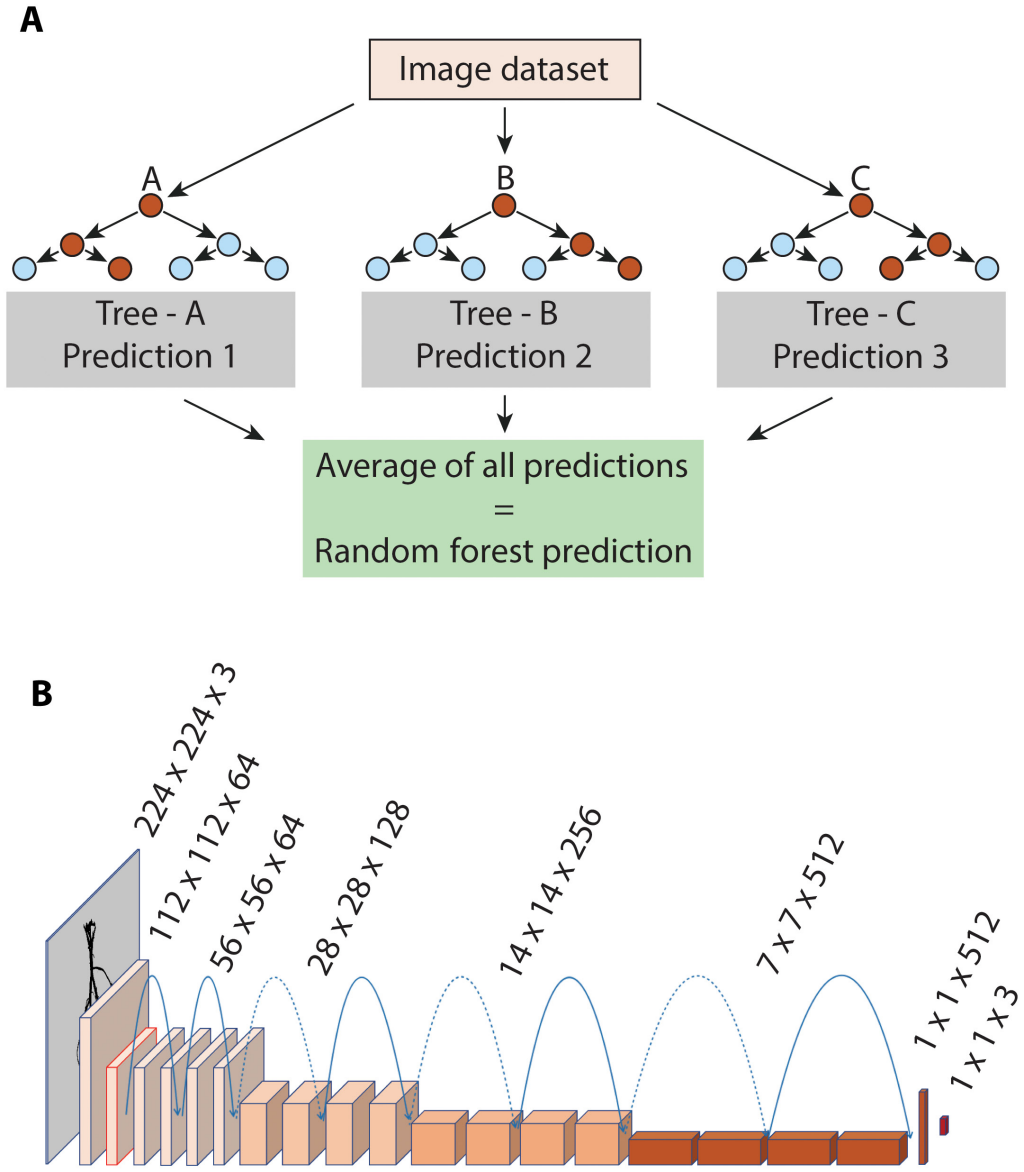
Simplified Random Forest and ResNet-18 model architectures. (A) Random Forest ML diagram. (B) ResNet-18 DNN model diagram. Solid blue lines represent identity shortcut connections of inputs and outputs of the same dimensions. Dotted blue lines represent identity shortcut connections of inputs and outputs of the different dimensions.

The 2 ML models, ResNet-18 (a CNN) and RF (a decision tree), were selected to train, test, and tune the 2 root crown datasets (see Fig. 5). These models were selected because they produced high accuracies in past research [11,32]. The RF model uses the extracted topological feature data from the scanned images while ResNet-18 uses the image data (pixels) for the analyses. Both models were trained and tested on the Oklahoma and Minnesota image datasets stratified into 3 classes (taproot [T], branched root [B], and branching taproot—intermediate [TB]).

#### Model descriptions

The RF model is a supervised ML algorithm used to predict/classify root types from image feature data. The training data used to construct the multiple decision trees of the model were derived from root trait analysis (38 or 27 traits) performed in RhizoVision Explorer. The 2 RF classification parameters were (a) “*m*_try_”—number of randomly selected variables to construct the decision trees (6 variables), and (b) “*n*_tree_”—number of trees used to calculate accuracies and probabilities (500 trees) [11,32]. Model parameters used in this project mirror those applied in Xu et al. [11].

ResNet-18 is one of the 5 versions (18, 34, 50, 101, and 152) of the widely used and award-winning (first place in ILSVRC 2015 classification task, ImageNet detection 2015, ImageNet localization 2015, COCO detection 2015, and COCO segmentation 2015) Res-Net DNN model, proposed to accommodate training on networks substantially deeper than those used previously [33]. Residual networks include “identity shortcut connections” that can be used when the input and output are the same dimensions (solid lines in Fig. 5B). When the dimensions increase (dotted lines in Fig. 5B), the shortcut continues to carry out identity mapping, or the projection shortcut is used to match dimensions (done by 1 × 1 convolutions) [33]. The key element of ResNet-18 enables a shortcut to directly add the output of one layer to another layer by bypassing intermediate layers [33]. The use of ResNet-18 (pretrained on ImageNet) over other competing models (U-net, VGGNet, etc.) is justified in that ResNets are one of the most efficient neural network architectures having the ability to reduce overparameterization via eliminating the exploding/vanishing gradient descent problem of many CNNs. Additionally, the choice of the shallower ResNet-18 over other deeper networks (such as ResNet-34, -50, etc.) was a product of the fact that (a) smaller networks are computationally quicker and, more importantly, that (b) it has been shown that smaller models can outperform their larger counterparts because larger/deeper models include more systematic label errors in their predictions than models with fewer parameters when tested on corrected labels for originally mislabeled examples [23].

Both models were trained and validated on an Ubuntu (version 18.04.5 LTS) server with 256 GB of memory and the server CPU was an Intel Xeon E5-2698. The training process was accelerated using a single NVIDIA GPU (Tesla V100 DGXS, 32 GB memory). Training for each model/label combination (original or corrected) was performed using the standard testing and training percentages (20% and 80%, respectively) for random 5-fold cross-validation.

#### Dataset and model combinations

**Fig. 6.**
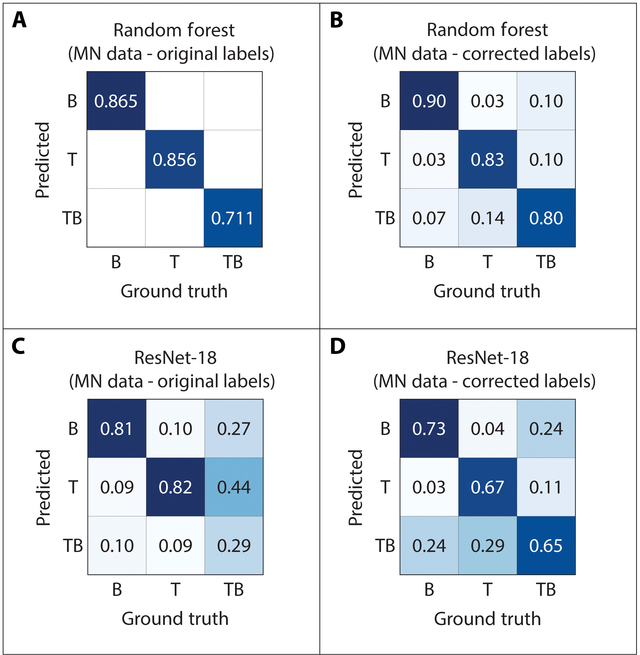
Random Forest and ResNet-18 confusion matrices derived from the MN dataset. (A) RF prediction accuracies from Xu et al. [11] (blank/misclassified values were not reported in Xu et al. [11]) (Table 1 cross reference = ID#1). (B) RF confusion matrix from corrected MN labels (Table 1 cross reference = ID#2). (C) ResNet-18 confusion matrix from original MN labels (Table 1 cross reference = ID#3). (D) ResNet-18 confusion matrix from corrected MN labels (Table 1 cross reference = ID#4).

**Fig. 7.**
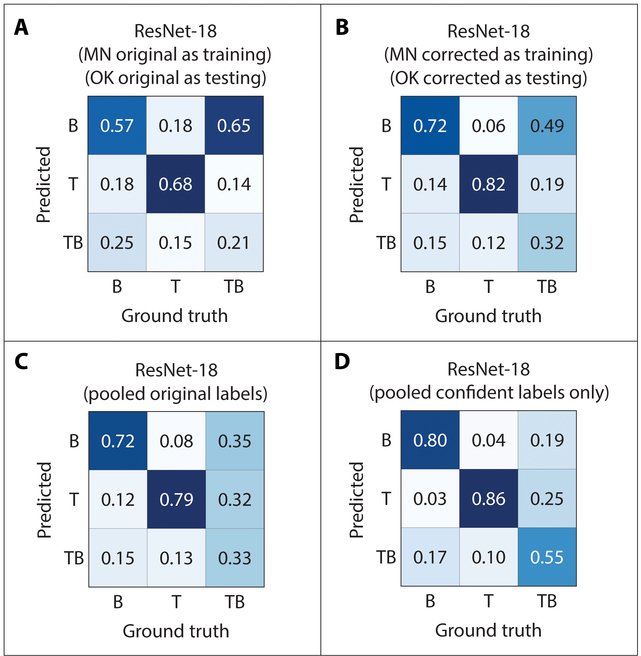
ResNet-18 confusion matrices derived from cross-population and pooled analyses. (A) ResNet-18 confusion matrix from original (MN and OK) labels (Table 1 cross reference = ID#5). (B) ResNet-18 confusion matrix from corrected (MN and OK) labels (Table 1 cross reference = ID#10). (C) ResNet-18 confusion matrix from pooled (*n* = 881) labels (Table 1 cross reference = ID#13). (D) ResNet-18 confusion matrix from pooled confident (*n* = 608) labels (Table 1 cross reference = ID#14). Additional matrices not shown here but discussed in Table 1 are included in the Supplementary Materials.

There were 3 ways in which the datasets from this study were combined and then tested using either the RF or ResNet-18 models. The first combination included only the Minnesota dataset (corrected or original labels) paired with RF or ResNet-18 (Table 1, rows 1 to 4; Fig. 6). The second type of dataset/model combinations included the 8 possible permutations of 2-fold cross-validations using the ResNet-18 model, where one dataset was used for training and the other is used for testing (8 separate cross-population validations) (see Fig. 7 and supplementary figures). In each fold, ResNet-18 was trained separately using training data both before and after label corrections with the RL and CL methods and then tested in a different fold with original labels. The final dataset/model combination tested in this study involved pooling both the Minnesota and Oklahoma datasets together, then applying the ResNet-18 model to the combined datasets (CL [*n* = 608] or no CL [*n* = 881]).

**Table 1.** Model prediction accuracies. Overall accuracy calculated using least squaremeans of the 3 RSA values. Highest individual class accuracies for each model are in boldface (RF or ResNet-18). Level I analysis refers to model outputs from independent datasets with no image label corrections; Level II analysis refers to cross-population model outputs using various combinations of corrected labels; Level III analysis refers to the use of various combinations of pooled data from both datasets (all original labels, or high-confidence labels only).

ID#	Input	Model and dataset combinations	Training data	Testing data	Branching (B)	Taproot (T)	Intermediate (TB)	Overall accuracy	CL applied	RL applied	Analysis level
**1**	Feature data (38)	Random forest—current study—MN data (*n* = 617)	MN original labels	MN original labels	0.856	**0.856**	0.711	0.828	No	No	I
**2**	Feature data (38)	Random forest—MN data only (*n* = 617)	MN-corrected labels	MN-corrected labels	**0.9**	0.83	**0.8**	0.838	Yes	Yes	II
**3**	Image	ResNet-18—MN data (*n* = 617)	MN original labels	MN original labels	0.81	0.82	0.29	0.700	No	No	I
**4**	Image	ResNet-18—MN data (*n* = 617)	MN-corrected labels	MN-corrected labels	0.73	0.67	**0.65**	0.679	Yes	Yes	II
**5**	Image	ResNet-18—cross-population MN (*n* = 617) and OK (*n* = 264)	MN original labels	OK original labels	0.57	0.68	0.21	0.447	No	No	I
**6**	Image	ResNet-18—cross-population MN (*n* = 617) and OK (*n* = 264)	OK original labels	MN original labels	0.58	0.53	0.27	0.492	No	No	I
**7**	Image	ResNet-18—cross-population MN (*n* = 617) and OK (*n* = 264)	MN-corrected labels	OK original labels	0.73	0.55	0.17	0.458	Yes	Yes	II
**8**	Image	ResNet-18—cross-population MN (*n* = 617) and OK (*n* = 264)	MN original labels	OK-corrected labels	**0.83**	0.57	0.06	0.491	Yes	Yes	II
**9**	Image	ResNet-18—cross-population MN (*n* = 617) and OK (*n* = 264)	OK-corrected labels	MN-corrected labels	0.54	0.85	0.24	0.556	Yes	Yes	II
**10**	Image	ResNet-18—cross-population MN (*n* = 617) and OK (*n* = 264)	MN-corrected labels	OK-corrected labels	0.72	0.82	0.32	0.576	Yes	Yes	II
**11**	Image	ResNet-18—cross-population MN (*n* = 617) and OK (*n* = 264)	OK original labels	MN-corrected labels	0.65	0.55	0.31	0.480	Yes	Yes	II
**12**	Image	ResNet-18—cross-population MN (*n* = 617) and OK (*n* = 264)	OK-corrected labels	MN-corrected labels	0.54	0.85	0.24	0.513	Yes	Yes	II
**13**	Image	ResNet-18—combined MN and OK data (*n* = 881)	Pooled original labels	Pooled original labels	0.72	0.79	0.33	0.637	Yes	No	III
**14**	Image	ResNet-18—combined MN and OK data (*n* = 608)	Confident pooled original labels	Confident pooled original labels	0.8	**0.86**	0.55	**0.748**	Yes	No	III

#### Image label corrections

Image labels were previously generated for the Minnesota data [11], then a portion (120) was adjusted via RL by 3 experienced researchers for the current study. The methods of RL involve inputting the originally labeled images into the model, then quantifying the accuracy of the model on the testing data. Images that were classified incorrectly during the testing phase are then manually relabeled and the model is tested again with the corrected image labels. The 2 accuracies (original and reactive) of the testing dataset can then be compared. CL was also applied to both image datasets in series with reactive learning RL methods (Fig. 3).

The CL method requires 2 inputs: (a) noisy labels (the original labels) and (b) predicted probability outputs from a model (softmax outputs for neural networks). The process of CL quantifies the model confidence of label accuracy by each class (the class’s probability threshold), which allows the individual labels to be evaluated as above or below each class threshold of the model and thereby produces a measure of the label’s confidence of correctness for each class in the model [22]. In general, CL involves 3 stages to characterize label errors: (a) pruning noisy data (searching for data that are mislabeled), (b) counting with probabilistic thresholds to estimate noise and avoid error propagation in learned model weights from reweighting the loss (training on clean data), and (c) ranking which data to use during training to allow learning with unnormalized probabilities or decision boundary distances (training with confidence) [22]. The label corrections were carried out using the *Cleanlab* package (https://github.com/cleanlab/cleanlab), along with root topology data, and RF as the classification model. This approach involved identifying error labels, relabeling, and cyclically retraining the model. Initially, the RF model was trained using original labels within the CL framework, assigning ranks and predicted labels to each training image. Images with predicted labels that did not match the original labels were manually reviewed, and root type labels for certain RSA images were revised. Using the updated labels, the RF model was retrained, and the CL framework continued to evaluate data quality until the RF model’s classification accuracy surpassed 0.8. A principal component analysis (PCA) was utilized on root topology data to observe the relabeling process.

#### Three levels of analysis

RSA images from 2 separate experiments were used to train and test the ResNet-18 and RF models. Both image datasets were independently evaluated using 3 levels of analysis (levels I to III) that employed the ResNet-18 model using a 5-fold cross-validation with and without label corrections by CL and RL. Level I analyses involved pairing either model (RF or ResNet-18) with uncorrected image labels (no CL or RL). Level II analyses involved pairing either model with intra- or cross-population data having corrected image labels, while level III analyses involved pairing pooled data (Minnesota + Oklahoma) with only the ResNet-18 model and while only applying CL (without RL) to the datasets to identify confident labels and/or stratify the pooled data prior to level III model executions. For each model/dataset/level test, confusion matrices were created to count the number of true positives (TP), true negatives (TN), false positives (FP), and false negatives (FN) for each permutation of the training/testing and image correction/original combinations between datasets. Each category in the confusion matrices were normalized by dividing the total number of images in each class, converting the counts number into percentages (Figs. 6 and 7 and Table 1). During each training round, the total number of TP predictions was summed to calculate the overall classification accuracy. The following sections describe the model and dataset combinations tested, along with their results.

## Results

### Intrapopulation prediction accuracies and label corrections

The intrapopulation (Minnesota data) prediction accuracies of the RF model without applying label confidence estimates or corrections using RL and CL (level I) were 0.865, 0.856, and 0.711 for B, T, and TB, respectively (Fig. 6A). The prediction accuracies of the ResNet-18 model (Minnesota data) without applying label confidence estimates or corrections using RL and CL (level I) were 0.810, 0.820, and 0.290 for B, T, and TB, respectively (Fig. 6C). Overall accuracies for the RF and ResNet-18 models using only Minnesota data and without applying label confidence estimates or corrections as input are 0.828 and 0.700, respectively. After applying RL and CL methods to identify and correct label errors (level II; Minnesota data only), some RSA class prediction accuracies increased, with corrected TB accuracy improving the most from 0.290 to 0.650 in the ResNet-18 model while TB accuracy also increased from 0.711 to 0.800 in the RF model. B accuracy modestly increased from 0.865 to 0.90 in the RF model, while B decreased in accuracy from 0.810 to 0.730 in the ResNet-18 model (Minnesota data only) (see Table 1). Level II T accuracy for the RF model decreased from 0.856 to 0.830, as did the ResNet-18 model accuracy from 0.82 to 0.67. Overall accuracies for the RF and ResNet-18 models using only MN data and with applying label confidence estimates or corrections as input are 0.838 and 0.679, respectively. These overall accuracy results from level II analyses indicate a minor (1%) increase in overall accuracy of the RF model and a decrease (2.1%) in overall accuracy for the ResNet-18 model. The percentages of images with corrected labels for each dataset were 31.6% (195 of 617 images) for the Minnesota dataset and 29.5% (78 of 264 images) for the Oklahoma dataset (see Table 2).

**Table 2.** Original and corrected label prediction accuracies of the RF model

	MN dataset (617 images)	OK dataset (264 images)
Original label accuracy	0.65	0.64
Iteration times	6	4
First-round error predicted	150	87
Last-round error predicted	33	15
Total corrected label number	195/617	78/264
Corrected label accuracy	0.85	0.83

### Cross-population prediction accuracies and label corrections

When the 2 datasets were compared using the 8 combinations of original versus corrected labels and as training versus testing datasets, the resultant confusion matrices indicated that, overall, the best cross-population data and training combination was corrected data (both datasets) and the use of Minnesota data to train the model to test on Oklahoma data. This matrix (Fig. 7B) has the second highest accuracy (82%) for predicting taproot (T) RSA out of the other combinations. For the intermediate RSA (TB), however, the prediction accuracy is relatively low, at only 32% (Fig. 7B) compared to the other root types. The branched RSA (B) prediction accuracy for corrected labels with Minnesota data used to train the model was not as high (72%) (Fig. 7B) as when original Minnesota labels were used to train the model on corrected Oklahoma data (83%) (Supplementary Figure ID#8). However, this combination also produced the highest overall accuracy within the cross-population class of analyses at 0.576. These results indicate that dataset prediction accuracies (by individual class or overall) can be improved with both RL and CL label corrections as well as image augmentation.

### Pooled data prediction accuracies

The final level of analysis (level III) involved running the ResNet-18 model using pooled datasets (Minnesota and Oklahoma) with their original labels (*n* = 881) and a model execution that applied CL to the pooled dataset to identify and use only the high confidence labels (*n* = 608) for testing and training. The prediction accuracies for these level III model outputs were among the highest of this study with B, T, and TB RSAs being 0.72, 0.79, and 0.33 for the pooled original labels (*n* = 881), respectively (Fig. 7C), and 0.80, 0.86, and 0.550 for B, T, and TB, respectively, of RSAs for the pooled original labels with high confidence (*n* = 608) (Fig. 7D). This final combination produced the highest overall prediction accuracy (0.748) (see Table 1, last row) of all the model/data combinations that employed ResNet-18 and demonstrates the gains achieved (~11%) by data pruning steps within the CL method. These various model prediction improvements (stratified by the final 2 levels of analysis) were noteworthy and confirm that applying either RL or CL methods is a viable technique to improve prediction accuracies.

### PCA of topology data

**Fig. 8.**
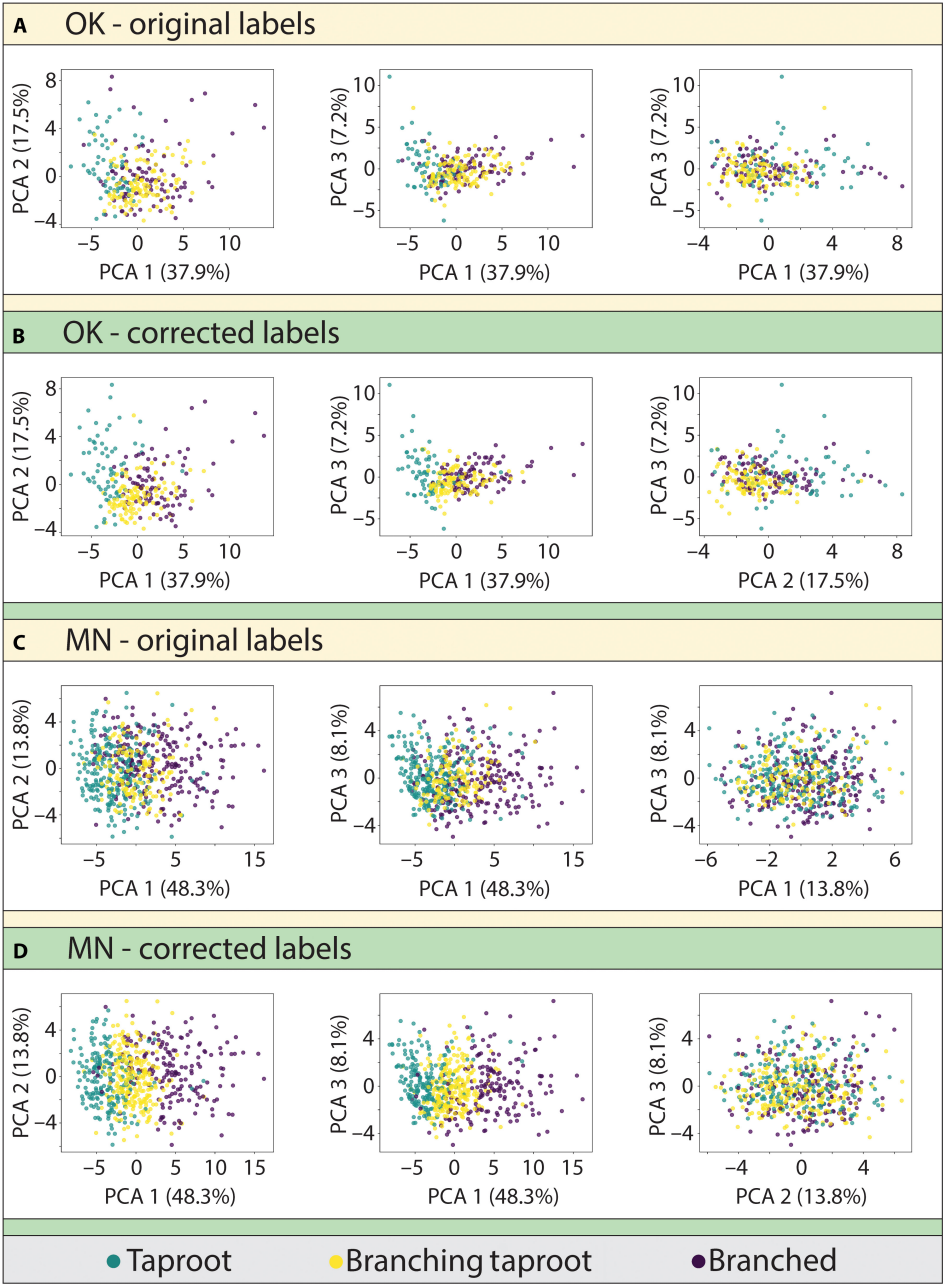
Label correction visualization using principal component analysis (PCA). Top rows (A and B) represent the 264 images from the Oklahoma dataset. Bottom rows (C and D) represent the 617 images from the Minnesota dataset.

**Fig. 9.**
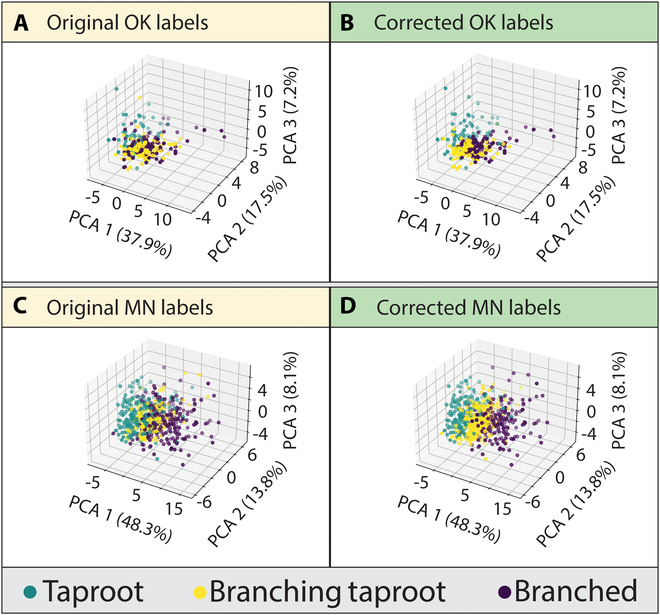
Three-dimensional PCA diagrams visualizing label corrections with topology data and RF model. Top row (A and B) represents the 264 images from the Oklahoma dataset. Bottom row (C and D) represents the 617 images from the Minnesota dataset.

Using topology data, PCA was used to observe the image label correction process. Label corrections resulted in reducing the dimensionality of the data and provided separability (clustering) between RSAs within the graphed output (Figs. 8 and 9). Though there was some overlap among RSA images, the corrected label graphs (especially with PCA1) showed close clustering of each RSA class, with the B RSA class being more dispersed than T or TB RSA classes. Figures 8 and 9 display the percentages of variance for the first 3 principal components of each dataset (*X*/*Y* axes).

### Image augmentation

Image augmentation was successfully implemented in the Minnesota dataset, which resulted in accuracy improvements for the model predictions. For the ResNet-18 model, both the B and T RSA prediction accuracies improved by 2% (B from 0.77 to 0.79; and T from 0.79 to 0.81) and the TB RSA had the most accuracy improvement at 11% (from 0.27 to 0.38) for the Minnesota dataset. Overall prediction accuracy for the ResNet-18 model improved from ~67% to ~71% when image augmentation was applied.

## Discussion

This study illustrates the challenges in labeling that lead to erroneous classifications and prediction accuracies related to differentiating RSAs based on ML techniques because of overfitting from very small training datasets or subtle differences in root architectures. Methods such as RL offer small increases in accuracy; however, there is a limit to the effectiveness and/or experimental bias incorporated into results when this method is over-applied or used as a tool to tune the model parameters via labeling. The manual labor or eyes-on requirements of RL are a limiting factor as the dataset size increases. This study involved RL with 881 original root images augmented 10 times to increase the dataset size (8,810), making manual image label corrections manageable, yet time-consuming. For large datasets containing millions of images, manual RL methods are not practical. CL is a partial solution to the RL bottleneck in that it is automated and because it gives weights to image labels based on predefined parameters in the CL algorithm by calculating label confidence using individual class thresholds.

### Label corrections, data augmentation, and overfitting

#### Image labeling

The manual labeling of alfalfa roots for this study highlights the difficulty related to the decision process or method used by each individual image labeler. Each individual perspective about root structures, morphology, or distribution used to differentiate between the 3 different root types can vary, which resulted in discrepancies between labels and classes. For this reason, classifying RSA based on quantitative values related to root morphology, topology, and distributions is critical to furthering image-based AI-driven phenotyping and related research. Arguments championing quantitative trait measurements over classifications may involve downstream interests such as trait interaction and/or how traits contribute to performance when continuous variables such as plant traits can be correlated to a desired outcome (i.e., yield or plant survival rates). A partial bridge between an automated AI classification analysis of RSA and the current feature extraction pipeline(s) is to convert continuous variables into classes that are based on numerical thresholds. Unfortunately, while binning these data, the resolution of a dataset is reduced from ratio to ordinal or interval scale data. However, this is necessary for at least 3 reasons related to this research: (a) discriminative AI models such as ResNet-18 require labels as input; (b) stakeholders such as alfalfa farmers often rely on classifications or ordinal scales to select desired cultivars with classified traits such as branch roots/taproots, pest/disease resistance or susceptibility, and winter survival [34,35]; and (c) breeders tend to be interested in the extremes that they can select into or out of a plant’s genetics via phenotyping, and in so doing produce 3 class distributions (high, medium, and low) by default with many traits. While still supervised by humans, current AI-based RSA image analyses do attempt to reduce human errors by sufficiently training a model to then produce its own results autonomously. In this respect, breeding efforts that utilize AI-based RSA image analysis help to minimize or remove human bias in the labeling process and are pushed further toward objective phenotyping [11].

Reactive learning can be used as an outside-the-model supervision strategy to improve model prediction accuracy; however, Lin and Weld [21] caution that models have varying levels of inductive bias. Weak inductive bias can cause low accuracy and overfitting due to noise in that as inductive bias weakens, overfitting increases and allows for the wrong data (noise) to be overfitted in the model. For classifiers with weak inductive bias, relabeling provides the most benefit because it reduces the likelihood of the model to overfit the noise [21]. In the case of misclassified RSA predictions, RL can be applied to a model dataset’s labeling errors identified via the preliminary executions of the model. By correcting the identified label errors, the model prediction accuracy on new image data is improved. Nevertheless, RL still needs human supervision of and input into its processes by observing and correcting model performance via image labels and relabeling. These eyes-on tasks lead to declines in efficiency compared to models that do not require human input or intervention [36]. Additionally, as the dataset size increases and as the number of label errors increases, there is a deleterious effect on throughput because of the manual labor required by RL to correct image label errors. For this study, a manageable 881 images were used to train, test, and validate separate models using label corrections indicated by preliminary model results. However, scaling this project up by many magnitudes to millions of images can cause RL to be impractical when time, cost, or labor constraints are relevant or limiting factors.

Confident learning offers a partial solution to the problem of image label errors in large datasets in that it automates the identification and estimation of class-conditional label noise (label errors), filters out the noisy labels, and then identifies and thereby allows the use of cleanly labeled data to train models with errors removed [22]. Another advantage of CL is that it is a model-agnostic family of theories and algorithms for characterizing, finding, and learning with label errors [22], thereby making it applicable to a variety of model architectures. This study has demonstrated that applying CL to noisy, image-direct inputs to models such as ResNet-18 can improve degraded (original) prediction accuracies as much as ~11% (pooled data + CL). Label noise is well-documented to undermine model training and evaluation. While many models can deal with image label noise, clean data produce better results when compared to the same data with its unreckoned noise left in [21,23,37]. In this study, commensurate gains in prediction accuracies were achieved when the datasets were cleansed with RL informed by CL methods, which was also seen in another study that compared cleansed and uncleansed labels that increased accuracies between ~1% and 6% [37].

The various combinations of (a) models and datasets and (b) testing and training datasets from this study show that modest accuracy improvements can be achieved by employing label correction methods to datasets (Table 1). The main objective of this study was to test the ability of CL and RL label corrections to improve the prediction accuracies of the ResNet-18 pixels-as-input model and quantify those potential improvements. By applying RL and CL to the datasets, the overall prediction accuracies increased by ~13% (cross-population vs. corrected cross-population) and ~17% (corrected cross-population vs. CL pooled) compared to the original label dataset (Table 1). A caveat to one ResNet-18 result (Minnesota data only) is that while overall accuracy did improve with label corrections, it came at a cost to one or more of the individual class prediction accuracies. For example, the corrected branching (B) and taproot (T) RSA class prediction accuracies decreased (8% and 15%, respectively) because of label corrections, but the intermediate class (TB) dramatically improved by 36%. This result indicates that the effect of label corrections on the ResNet-18 pixels-as-input model increases the ability of the model to predict intermediate RSAs, which incidentally are the most difficult to classify because of shared traits with the other classes. When the Oklahoma and Minnesota datasets are pooled and original labels are used to train and test the ResNet-18 model, both the individual RSA classes and overall prediction accuracies are dramatically improved by applying CL (~8% to 22% for individual classes and ~11% overall). These results demonstrate that the RSA prediction accuracy of the ResNet-18 is improved with RL and/or CL when using images as direct inputs into the model. Although the ResNet-18 model did have lower overall accuracy (or accuracies) than the RF model, the increase in the model’s ability to distinguish between the intermediate class (TB) by employing RL and CL is promising and warrants further investigations with new datasets in the future. Furthermore, while the RF model did have better overall and individual RSA class accuracies than most of the ResNet-18 model results, these higher accuracies may result from the inherent bias due to human error within the RF model from using feature data as inputs. This bias problem can be circumvented by using an image-direct model such as ResNet-18.

#### Prediction accuracies, overfitting, and other sources of error

To address and prevent overfitting issues, cross-population validation between the Minnesota and Oklahoma datasets was tested in various combinations (Table 1). The highest overall accuracy combination of all the cross-population validations was the corrected Minnesota data used as training data and corrected Oklahoma data as the testing data.

Low prediction accuracy found during the cross-population validation could be a result of the relatively small overall dataset size, which is a known cause of overfitting [38], or the ratio between training and testing data that did not follow the traditional 80% and 20% convention (Oklahoma testing = ~30%, Minnesota training= ~70%). Additionally, the low prediction accuracy for the intermediate TB RSA class (~32%) might be explained by the fact that the TB RSA inherently contains traits from both the T and B classes and therefore confounds the model algorithm when asked to differentiate images of the TB class from both the T and B classes. Anecdotally, expert manual labeling can also be plagued by the same issue, perhaps for similar reasons; the extremes of an ideotype are more obvious and easier to classify than the average, intermediate, or mixed RSA image features. A further confounding issue with roots is these plants were grown in different locations with major differences in soil, climate, and especially plant health. Therefore, it is possible that the generally low cross-population prediction accuracies (no overall accuracy over 58% for any permutation) could have been caused by environmental differences that manifested within the sample imagery, for example, a disease causing some Oklahoma plants to lose their taproots, or soil types and/or properties that variously hindered or promoted root growth. The gains in accuracy achieved by pooling the Oklahoma and Minnesota datasets indicate that the variability of RSAs between different populations can be mitigated when the ML model is trained using a representative sample set from all populations.

Another possible reason for the low prediction accuracies for the TB RSA class in many of the confusion matrices could be from genetic differences between Minnesota and Oklahoma data that control morphological, topological, and distributional differences. The differences of root phenotype states could introduce uncertainty or error into the model because of inherent genetic RSA differences between populations. The model could not accurately predict RSA as well during cross-population validations as it did with intrapopulation validations or pooled cross-validations. When the Minnesota data alone were used to train and test the ResNet-18 model, the predicted accuracies of B, T, and TB RSAs using corrected data labels were 73%, 67%, and 65%, respectively. Conversely, the cross-population validation using Minnesota as training data (Oklahoma testing data) predicted that accuracies of B, T, and TB RSAs using corrected data labels were 72%, 82%, and 32%, respectively. For the pooled dataset (*n* = 608) that only involved high-confidence original labels, predicted accuracies of B, T, and TB RSAs were 80%, 86% (the highest prediction accuracy for this class), and 55%, respectively. These results may indicate that RSA differences between populations are partially responsible for low TB RSA prediction accuracies after label corrections and data augmentation were employed. Additionally, the TB class does not achieve as high of a prediction accuracy in the various permutations of cross-population data combinations as it does in the MN-corrected (training) and OK-corrected (testing) permutation (Table 1, ID#10). The same is true with the B class in that permutation as well, with the caveat that permutations ID#7 and ID#8 are slightly higher but fall short in the T class at 55% and 57%, respectively. It is possible that because the MN dataset was bred for specific RSAs (branch and tap), the model may have been better able to deal with the OK dataset, which is known to have health problems that likely perturbed the model performance. Because the MN root images are of more refined ideotypes (fourth cycle of breeding) than the OK root images, the model may have been better equipped to classify them, whereas using corrected OK data to train and MN data to test had an opposite effect and led to lower prediction accuracies.

#### Image data as direct input to models

Imagery-based RSA research that relies on images as direct input to AI models is one approach that appears to have promise to improve throughput. Based on the findings of this research, competing AI models using extracted features instead of pixel data as inputs have a lower throughput (i.e., simpler model process = higher throughput). Because throughput measures rates by units over time, the proposed iterative and self-correcting method that combines image analysis and label correction strategies is markedly faster than traditional methods. As an example, during the creation of this manuscript, ResNet-50 was installed on an alpha version mobile application (App) that can take a raw image from a device’s camera as input and then run ResNet-50 on that image and produce a classification and its prediction probability near instantaneously. The manual equivalent (human-derived label) for some roots can be several minutes because of root counts, topology decisions, or size estimations. When considering that batch processing on supercomputers is then leveraged on entire root datasets, the argument for “faster than humans” is easily made with this new in silico method (though we did not explicitly measure time gains between methods for this study). Additionally, image-direct methods contain less bias from human input, and this is evidenced by the notable improvements in prediction accuracies after CL and RL are applied. This study has highlighted the need for less manual labor and more automation of image analysis pipeline tasks and the need for clean data. Even though AI models such as ResNet-18 are quite capable of accurately predicting properly (cleanly) labeled and adequately sized image dataset RSAs, no model can properly deal with mislabeled training data. Image data directly input into phenotyping models is one more step toward automated objective phenotyping that is aimed at removing human bias and/or error from AI phenotyping tasks. However, image data as direct input into AI models to detect, classify, or predict RSA cannot fully remove the inherent biases caused by image capture and labeling to train the model during the model development and optimization stage. This type of bias or even error is evidenced by the variations in label decisions regarding RSA classes in this study between expert labelers. One major advantage of the image-direct approach to modeling RSA is that many tasks like classification, which completely rely on human inputs, are now in silico and only require a modicum of supervision to perform the same work faster, more consistent across researchers and labs, and more accurately.

### Final thoughts

This study confirmed the feasibility of using RSA images (pixels) as direct inputs into a DNN as a suitable and less error-prone replacement to the more traditional method of feeding extracted RSA feature data into an AI model, such as RF. Additionally, this study highlights the moderate abilities of 2 separate label correction strategies to improve model prediction accuracies of alfalfa RSA images in low-cost and time-efficient ways that improve performance and reduce overparameterization from outside the model. However, while taproot-dominant RSAs and branched-dominant RSAs are easily managed by our models (predicted with high accuracies; over 80%), the intermediate-type RSAs were not (predicted with low accuracies; below 66%). Therefore, further development of algorithms that address intermediate root features via image-direct model input is needed. Lastly, this research and its use of shared image datasets from other authors illustrate the importance and benefits of an “open science” approach where data can be reused and applied to future questions and problem-solving.

## Data Availability

The original images (dataset 1 from USDA-ARS at St Paul, MN) with tags removed and segmented images from RootPainter for data analysis are available on Zenodo (https://doi.org/10.5281/zenodo.5879778). Dataset 2 from Oklahoma: Root crown images and R statistical analysis code generated from this study are available on Zenodo (https://doi.org/10.5281/zenodo.2172832). All the data used in this study are available per request from the corresponding authors.
