# Supplementary material for: Phenotyping Alfalfa (Medicago sativa L.) Root Structure Architecture via Integrating Confident Machine Learning with ResNet-18
Source: Plant Phenomics. 2024 Sep 11;6:0251. doi: 10.34133/plantphenomics.0251 (PMC11387747; doi:10.34133/plantphenomics.0251)
Supplement: Supplementary 1 — Figs. S1 to S7 [file plantphenomics.0251.f1.docx]

**Supplemental Materials**

**
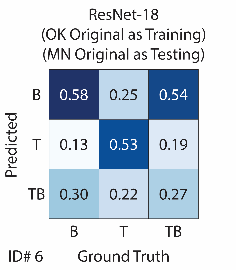

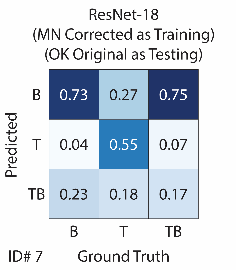

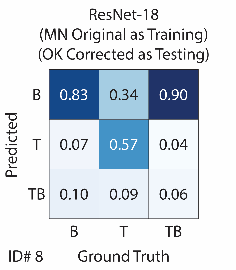

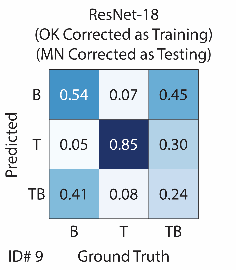
**

**
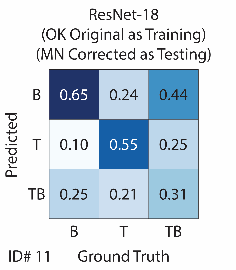

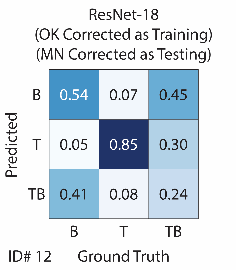
**

Supplemental Figs. 1-6. Uncited confusion matrices. ID# in figures refer to Table 1 (column 1) rows of the same name.

**
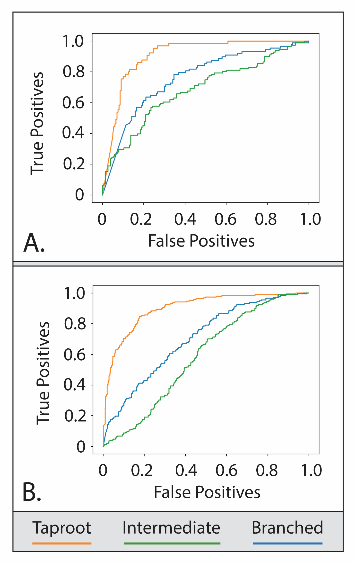
**

Supplemental Fig. 7. Receiver operator characteristic (ROC) curves. A) ROC curve when using MN data as training set during cross validation. B) ROC curve when using OK data as training set during cross validation.
